# Fasting Reveals the Effects of a Plant Extract‐ and Microalgae‐Derived Nutraceutical on Lipid Metabolism and Hepatic Physiology in Juvenile *Sparus aurata* Fed a Plant‐Based Diet

**DOI:** 10.1155/anu/9345086

**Published:** 2026-05-19

**Authors:** Anyell Caderno, Milagrosa Oliva, Alba Galafat, Antonio Astola, Francisco Javier Alarcón-López, Juan Miguel Mancera, Juan Antonio Martos-Sitcha

**Affiliations:** ^1^ Department of Biology, Faculty of Marine and Environmental Sciences, International Campus of Excellence of the Sea (CEI·MAR), University Marine Research Institute (INMAR), University of Cádiz, Puerto Real, 11519, Spain, uca.es; ^2^ Department of Biology and Geology, Faculty of Experimental Sciences, International Campus of Excellence of the Sea (CEI·MAR), University of Almería, Almería, 04120, Spain, ual.es; ^3^ Department of Biomedicine, Biotechnology and Public Health, Faculty of Sciences, University of Cádiz, Puerto Real, 11519, Spain, uca.es; ^4^ Lifebioencapsulation S.L., Parque Científico PITA, Almería, 04131, Spain

**Keywords:** feed deprivation, functional aquafeeds, gilthead seabream, hepatoprotection, nutritional resilience, nutritional stress

## Abstract

This study investigated the effects of a plant‐based diet supplemented with a nutraceutical derived from plant extracts and microalgae on hepatic function and lipid metabolism during a fasting challenge. Juvenile *Sparus aurata* were fed three experimental diets for 90 days, followed by 14 days of feed deprivation. The diets consisted of: (i) a control formulation representative of commercial feeds for the species (C+: 20% fishmeal [FM], 9% fish oil [FO]); (ii) a low‐FM, predominantly plant‐based diet containing 5% FM and 5% FO (C‒); and (iii) the same C‒ diet supplemented with 1% *LB-LIVERprotect* nutraceutical (LP). LP supplementation improved specific aspects of fatty acid composition in mesenteric fat, including eicosapentaenoic acid (EPA) content and the omega‐3/omega‐6 ratio. These changes were potentially associated with increased hepatic expression of desaturases (*fads2* and *scd1a*). Fish in the LP group also exhibited a higher mesenteric index (MSI), together with hepatic upregulation of key genes involved in lipid catabolism (*lpl*, *hl*, *atgl*, and *hsl*). Following feed deprivation, a marked reduction in both MSI and the expression of lipid catabolic genes supported active mobilization of mesenteric fat in LP‐fed fish. This response was accompanied by signs of hepatic protection during fasting, including preserved energy reserves, a higher hepatosomatic index (HSI), and reduced hepatocyte atrophy. Moreover, LP supplementation partially attenuated several adverse effects observed in the C‒ group, such as elevated plasma cortisol levels and increased indicators of energy demand during fasting. Consistently, LP‐fed fish showed fewer signs of fasting‐induced liver damage, as reflected by an approximately 3.4‐fold lower plasma alanine aminotransferase (ALT) activity compared to the C‒ group. Overall, *LB-LIVERprotect* shows potential to support fish health and tissue integrity during feed deprivation and to mitigate some of the negative effects associated with plant‐based aquafeeds.

## 1. Introduction

The intensification of modern aquaculture has been accompanied by a progressive replacement of fishmeal (FM) and fish oil (FO) with plant protein sources (PP) and vegetable oils (VO) in commercial aquafeeds, driven by economic and sustainability constraints [1]. This shift has introduced complex nutritional challenges that extend beyond meeting basic dietary requirements, particularly in relation to metabolic regulation and physiological robustness under intensive rearing conditions [1, 2]. In response, functional feeds have been developed as an integrated nutritional strategy, in which ingredient selection and formulation are specifically designed to support key physiological processes and maintain performance when conventional nutritional paradigms are pushed to their limits [3–5]. Within this framework, nutraceuticals have emerged as a complementary approach, referring to food‐derived bioactive compounds formulated in concentrated preparations and incorporated into aquafeeds to enhance growth performance, immune competence, stress tolerance, and metabolic regulation [6–11].

Accordingly, there is growing interest in functional ingredients derived from plant by‐products and microalgae as sustainable and natural alternatives to synthetic compounds [12]. Plant extracts rich in phenolic compounds are particularly promising due to their antioxidant and metabolic regulatory properties [13]. Notably, grape marc, a by‐product of the wine industry, stands out for its high content of bioactive molecules, including flavonoids, phenolic acids, procyanidins, and resveratrol [14, 15]. These products offer additional advantages, such as an attractive nutritional profile, low cost, and their use contributes to promoting a circular economy [16]. Moreover, they have proven to be effective in aquaculture nutrition, yielding promising results [17, 18].

Microalgae are also widely used as functional ingredients and nutraceutical sources due to their high nutritional value [19], characterized by elevated protein and lipid levels and balanced amino acid profiles comparable to conventional plant and animal sources [20, 21]. They contain multiple bioactive compounds, including pigments, fatty acids (FA), and peptides, with antioxidant, anti‐inflammatory, and immunomodulatory properties [9, 22–24], and they naturally accumulate vitamins and minerals [25]. Consequently, the inclusion of microalgal‐based ingredients in aquafeeds is of growing interest for dietary fortification [12, 26]. In this context, microalgae have been employed to enhance the content of omega‐3 polyunsaturated fatty acids (n‐3 PUFA), such as eicosapentaenoic acid (EPA) and docosahexaenoic acid (DHA), and to improve the omega‐3/omega‐6 ratio (n‐3/n‐6) in tissue lipids [21, 27, 28]. This is particularly relevant because many fish species have limited endogenous capacity to synthesize these FA, and inadequate supply can impair multiple physiological processes [29]. Therefore, essential FA must be provided through the diet [24].

Carnivorous fish species are metabolically adapted to diets rich in protein and lipids and low in carbohydrates [30], a feature that makes them particularly sensitive to dietary formulations that deviate from this profile. A high inclusion of plant‐based ingredients can disrupt lipid metabolism and hepatic function [31, 32], often accompanied by reductions in tissue n‐3 PUFA levels, with potential consequences for growth performance and product quality [25, 28]. These metabolic disturbances can be further exacerbated by additional stressors such as feed deprivation or environmental stress [33], emphasizing the importance of nutritional strategies capable of maintaining metabolic homeostasis under demanding conditions.

Fasting is a frequent and physiologically relevant condition in both wild and farmed fish, occurring due to environmental fluctuations, reproductive cycles, handling, transport, preharvest management, or restricted feeding strategies used to control body composition and improve production efficiency [34–36]. During fasting, fish undergo a coordinated metabolic orchestration generally characterized by rapid depletion of hepatic glycogen followed by enhanced lipid mobilization and triacylglycerol lipolysis, supplying nonesterified FA for mitochondrial β‐oxidation. These adjustments primarily involve hepatic and adipose tissue metabolism and reflect a shift toward lipid‐based energy utilization under nutrient deprivation [37, 38]. Therefore, fasting represents a robust metabolic challenge model to evaluate nutritional strategies aimed at improving hepatic function and lipid metabolic resilience.

In the present study, juvenile gilthead seabream (*Sparus aurata*) were fed a diet rich in plant‐based ingredients supplemented with the nutraceutical *LB-LIVERprotect*, composed of plant extracts and a microalgal blend of enzymatically hydrolyzed *Arthrospira* sp. and *Nannochloropsis* sp. This nutraceutical was developed to support hepatic and renal function, promote essential phospholipid synthesis, and provide lipotropic activity associated with the prevention of fatty liver development. Its effects on growth, welfare, intermediary metabolism, and intestinal structure and functionality have been previously investigated by Caderno et al. [39] in *S. aurata* after 90 days of continuous feeding with the same plant‐based dietary formulation. However, its potential role in supporting hepatic and lipid metabolic responses under metabolic stress induced by nutritional challenges has not yet been evaluated. This is particularly relevant in modern aquaculture, where the increasing use of plant‐based diets requires nutritional strategies to ensure optimal growth, health, and welfare in high‐value species such as gilthead seabream. We therefore hypothesized that dietary supplementation with *LB-LIVERprotect* would enhance hepatic function and lipid metabolic resilience during fasting in fish previously fed low‐FM, plant‐based formulations. To test this hypothesis, the present study examined the effects of *LB-LIVERprotect* on lipid metabolism, essential FA composition in mesenteric fat, and liver health under fasting conditions following prolonged exposure to these formulations. Given the liver’s central role in lipid regulation and energy mobilization, the combined effects of plant‐derived nutrition and fasting may challenge the physiological limits of fish.

## 2. Materials and Methods

### 2.1. Ethics

Fish were kept and handled according to the guidelines for experimental procedures in animal research established by the Ethics and Animal Welfare Committee of the University of Cádiz, by Spanish laws (RD 53/2013 and RD 118/2021), and European Union legislation (2010/63/EU). The Ethical Committee of the Autonomous Government of Andalusia approved the experiments (Junta de Andalucía reference number 23/10/2019/176).

### 2.2. Diets and Feeding Protocol

Three isonitrogenous (43% crude protein) and isolipidic (17% crude lipid) diets were prepared (Tables 1 and 2) and produced at the University of Almería facilities (Experimental Feeds Service; https://www.ual.es/universidad/serviciosgenerales/stecnicos/perifericos-convenio/piensos-experimentales): (i) the control diet (C+), formulated with a nutritional profile based on FM and FO, closely resembling current commercial feeds for gilthead seabream (containing 20% FM and 9% FO); (ii) a second diet (C‒) formulated with only 5% FM and 5% FO, and a high content of PP and VO; and (iii) the C‒ diet supplemented with 1% (10 g kg^−1^ feed) of the nutraceutical compound *LB-LIVERprotect* (LP), provided by LifeBioencapsulation S.L. (Almería, Spain; https://lifebioencapsulation.com/). *LB-LIVERprotect* is a concentrated formulation consisting of 700 g kg^−1^ of artichoke and grape marc polyphenolic extracts, and choline salts, combined with 300 g kg^−1^ of a hydrolyzed microalgae blend composed of *Arthrospira* sp. and *Nannochloropsis* sp [39].

**Table 1:** Ingredients and proximate composition (% dry matter) of experimental diets for juvenile gilthead seabream (*Sparus aurata*).

Ingredients	C+	C‒	LP
Fishmeal LT94^a^	20.00	5.00	5.00
Lysine^b^	1.20	1.20	1.20
Methionine^c^	0.50	0.60	0.60
*LB-LIVERprotect* ^d^	—	—	1.00
Squid meal^e^	2.00	2.00	2.00
Fishmeal hydrolysate CPSP90^f^	1.00	1.00	1.00
Krill meal^g^	2.00	2.00	2.00
Wheat gluten^h^	8.00	11.00	11.00
Soybean protein concentrate^i^	26.00	36.00	36.00
Pea protein concentrate^j^	7.50	10.60	10.60
Fish oil^k^	9.00	5.00	5.00
Soybean oil^l^	4.30	10.10	10.10
Soybean lecithin^m^	1.00	1.00	1.00
Wheat meal^n^	12.90	9.60	8.60
Monocalcium phosphate^o^	0.50	0.80	0.80
Vitamin and mineral premix^p^	2.00	2.00	2.00
Vitamin *C* ^q^	0.10	0.10	0.10
Guar gum^r^	2.00	2.00	2.00

**Proximate composition (% dry matter)**			

Crude protein	43.10	43.10	42.80
Crude lipid	16.80	17.10	16.80
Ash	8.30	6.80	6.80

*Note:* Dietary codes: C+, positive control containing 20% FM and 9% FO; C‒, negative control containing 5% FM and 5% FO; LP, C‒ diet supplemented with 1% *LB-LIVERprotect*.

^a^Fishmeal LT94 (69.4% crude protein, 12.3% crude lipid; Norsildemel, Bergen, Norway).

^b,c^Lysine and methionine (Lorca Nutrición Animal S.A., Murcia, Spain).

^d^ *LB-LIVERprotect*: nutraceutical compound composed of 700 g kg^−1^ artichoke extract, grape marc polyphenolic extract, and choline salts, plus 300 g kg^−1^ of a hydrolyzed microalgae blend composed of *Arthrospira* sp. and *Nannochloropsis* sp. (LifeBioencapsulation S.L., Almería, Spain).

^e,f,g^Squid meal, fishmeal hydrolysate CPSP90, and krill meal (76%, 84%, and 56% crude protein, respectively; Bacarel, UK). CPSP90 corresponds to enzymatically predigested fishmeal.

^h^Wheat gluten (78% crude protein; Lorca Nutrición Animal S.A., Murcia, Spain).

^i^Soybean protein concentrate (Soycomil; 60% crude protein, 1.5% crude lipid; ADM, Poland).

^j^Pea protein concentrate (85% crude protein, 1.5% crude lipid; Emilio Peña S.A., Spain).

^k^Fish oil (AF117DHA; Afamsa, Spain).

^l^Soybean oil (Aceites El Niño, Spain).

^m^Soybean lecithin (P700IP; Lecico, Germany).

^n^Wheat meal (local providers, Almería, Spain).

^o^Monocalcium phosphate (Andrés Pintaluba, Spain).

^p^Lifebioencapsulation S.L. (Almería, Spain). Vitamins (mg kg^−1^): vitamin A (retinyl acetate), 2,000,000 IU; vitamin *D*_3_ (DL, cholecalciferol), 200,000 IU; vitamin E (Lutavit E50), 10,000 mg; vitamin K_3_ (menadione sodium bisulfite), 2500 mg; vitamin *B*_1_ (thiamine hydrochloride), 3000 mg; vitamin *B*_2_ (riboflavin), 3000 mg; calcium pantothenate, 10,000 mg; nicotinic acid, 20,000 mg; vitamin *B*_6_ (pyridoxine hydrochloride), 2000 mg; vitamin *B*_9_ (folic acid), 1500 mg; vitamin *B*_12_ (cyanocobalamin), 10 mg; vitamin H (biotin), 300 mg; inositol, 50,000 mg; betaine (Betafin S1), 50,000 mg. Minerals (mg kg^−1^): Co (cobalt carbonate), 65 mg; Cu (cupric sulfate), 900 mg; Fe (iron sulfate), 600 mg; I (potassium iodide), 50 mg; Mn (manganese oxide), 960 mg; Se (sodium selenite), 1 mg; Zn (zinc sulfate), 750 mg; Ca (calcium carbonate), 18.6% (186,000 mg); KCl, 2.41% (24,100 mg); NaCl, 4.0% (40,000 mg).

^q^Vitamin C (TECNOVIT, Spain).

^r^Guar gum (Emilio Peña S.A., Spain).

**Table 2:** Fatty acids profile (% of total fatty acids) of experimental diets for juvenile gilthead seabream (*Sparus aurata*).

Fatty acids	C+	C‒	LP
14:0	2.07	1.15	1.18
16:0	17.02	15.82	17.43
18:0	4.51	4.05	4.13
16:1n‐7	2.85	1.47	0.96
16:2n‐4	0.12	0.23	0.17
16:3n‐4	0.54	0.27	0.28
18:1n‐9	19.15	25.75	26.28
18:2n‐6	22.52	43.75	44.95
18:3n‐3	2.39	0.26	0.07
20:1n‐9	1.09	0.88	0.91
20:4n‐6	1.26	0.55	0.54
20:4n‐3	1.70	0.27	0.39
20:5n‐3	5.51	2.62	2.67
22:5n‐3	0.81	0.40	0.41
22:6n‐3	12.88	7.02	7.18
Other FA	4.97	2.27	2.07
ΣSFA	23.60	21.02	22.74
ΣMUFA	23.09	28.11	28.15
ΣPUFA	25.18	11.35	11.26
Σn‐3	23.92	12.84	10.72
Σn‐6	23.78	44.31	45.49
Σn‐9	1.09	0.88	0.91
n‐3/n‐6	1.01	0.30	0.24
EPA/DHA	0.43	0.37	0.37

*Note:* Dietary codes: C+, positive control containing 20% FM and 9% FO; C‒, negative control containing 5% FM and 5% FO; LP, C‒ diet supplemented with 1% *LB-LIVERprotect*, 10 g kg^−1^ feed.

Abbreviations: DHA, docosahexaenoic acid; EPA, eicosapentaenoic acid; FA, fatty acids; MUFA, monounsaturated fatty acids; PUFA, polyunsaturated fatty acids; SFA, saturated fatty acids. Additional details are provided in the legend of Table 1.

The trial was conducted at the Aquaculture Technological Center (CTAQUA; El Puerto de Santa María, Cádiz, Spain; Spanish Operational Code REGA ES110270000411). Fish were acclimated for 14 days and fed the control diet (C+). After this period, juveniles with an initial mean body mass of 28.40 ± 0.20 g were randomly distributed into nine 400 L tanks (~2.50 kg m^−3^ initial stocking density; *n* = 30 fish per tank; *n* = 90 fish per experimental group). Diets were assigned to tanks using a color‐coded randomization system, ensuring that replicate treatments were not placed in adjacent tanks. The feeding trial was performed as previously described by Caderno et al. [39]. Briefly, fish were fed aquafeeds to apparent satiety (*ad libitum*) twice daily, 6 days per week, over 90 days (September–December 2020). The trial was conducted in a blinded manner to minimize potential bias related to diet composition. Feed intake (FI) was recorded weekly for each replicate tank. The effects of the experimental diets on growth and different physiological processes in *S. aurata* have already been reported [39].

After the feeding trial, fish from each experimental group were lightly sedated with 2‐phenoxyethanol (0.4 mL L^−1^ seawater) and sampled for biometric measurements. Subsequently, fish were divided into two subgroups and subjected to different feeding regimes for an additional 14 days: (i) fasting (Ft group) and (ii) continued feeding with their respective experimental diets (Cf group). For this purpose, fish from each diet and tank were transferred to triplicate cylindrical enclosures made of rigid mesh and suspended within larger 3000 L tanks assigned to each feeding regime (Ft or Cf). Stocking density was maintained at levels equivalent to those of the original experimental tanks.

Previous studies have demonstrated that similar periods of feed restriction are sufficient to induce metabolic and physiological alterations and to disrupt homeostasis in *S. aurata* [40, 41]. In other marine teleosts, fasting durations of ~10 days have also been shown to promote lipid mobilization and metabolic disturbances [42]. In contrast, shorter fasting durations may be insufficient to elicit active lipid mobilization when fish are well nourished prior to feed deprivation. Accordingly, the selected fasting period was adequate to induce measurable physiological changes in juvenile *S. aurata*, allowing the assessment of their responses in relation to prior nutritional status and dietary supplementation strategies. At the same time, it avoided prolonged or excessive fasting that could have caused severe physiological stress and confounded data interpretation.

Throughout the experiment, fish were maintained in a recirculating aquaculture system (RAS) equipped with mechanical and biological filtration to ensure optimal water quality. The system also included programmable sensors for the continuous monitoring and control of temperature and dissolved oxygen, with one data point recorded every 15 min. Water temperature was maintained at 21–22°C, and salinity was manually checked daily to ensure a constant value of 36 ‰. Dissolved oxygen levels at the tank outlets remained above 85% saturation throughout the experiment. The photoperiod was adjusted to follow the natural daylight cycle at the study site (36°35^′^06″ N; 06°13^′^48″ W) during the experimental period.

### 2.3. Sampling Procedures

At the end of the feeding/fasting period (14 days), final sampling was carried out to record biometric parameters and collect biological samples. Fish under continuous feeding conditions were fasted for 24 h prior to the procedure. All procedures were performed in the morning, starting at 09:00 h. To minimize potential circadian effects, fish from all experimental groups were sampled alternately over the entire duration of the procedure (~2.5 h). A total of eight fish per dietary treatment (C+, C‒, and LP) and feeding condition (Cf or Ft) were randomly selected (2–3 fish per experimental triplicate), resulting in 48 fish overall. Fish were deeply anesthetized with a lethal dose of 2‐phenoxyethanol (1 mL L^−1^ seawater). Blood was withdrawn from the caudal vessels using heparinized syringes immediately after anesthesia. Plasma was obtained by centrifugation (13,000 × *g* for 4 min), snap‐frozen in liquid nitrogen, and kept at −80°C until biochemical and hormonal analyses. Following blood collection, fish were euthanized by cervical transection. The liver and perivisceral fat were dissected and weighed to calculate the hepatosomatic index (HSI) and mesenteric index (MSI), respectively. Tissues were snap‐frozen in liquid nitrogen and stored at −80°C for subsequent fatty acid and biochemical analyses. In addition, a small liver biopsy was collected from each fish for gene expression assessment and immediately preserved in RNAlater (Invitrogen, Thermo Fisher Scientific). Samples were maintained at 4°C for 24 h to allow complete reagent penetration and then stored at −20°C until total RNA extraction. Liver and anterior intestine samples for histological analysis were also collected from a subset of four fish previously used for biochemical and molecular analyses (1–2 fish per experimental triplicate). All histological samples were fixed in buffered formalin (10% formalin in phosphate‐buffered saline, pH 7.2) for 48 h.

### 2.4. Growth Performance and Biometric Parameters

The following equations were used to calculate growth performance and biometric indices: (i) Specific growth rate (SGR, % day^−1^) = 100 × [ln final body weight − ln initial body weight)/number of days]; (ii) Hepatosomatic index (HSI, %) = 100 × (liver weight/total body weight); and (iii) Mesenteric index (MSI, %) = 100 × (perivisceral fat weight/total body weight).

### 2.5. Experimental Procedures

#### 2.5.1. Biochemical Parameters

Plasma concentrations of glucose (Ref. 1001200), lactate (Ref. 1001330), triglycerides (TAG; Ref. 1001311), and cholesterol (Ref. 41021) were determined with commercial kits from SPINREACT S.A. (St. Esteve d′en Bas, Girona, Spain). Total plasma protein concentration was assessed using a bicinchoninic acid (BCA) assay kit (Pierce, Rockford, USA). All metabolite analyses were based on colorimetric reactions, in which the formation of a chromogenic product allows quantification via absorbance measurement. Concentrations were calculated using standard curves generated with kit‐specific calibrators of known values. Assays were adapted to a 96‐well microplate format, and all samples were analyzed in technical duplicate. Absorbance readings were obtained with a PowerWave 340 microplate spectrophotometer (Bio‐Tek Instruments, Winooski, VT, USA). Data were processed using KCjunior software (Microsoft‐compatible).

Frozen liver biopsies were mechanically homogenized in 7.5 volumes of ice‐cold 0.6 N HClO_4_ using a blender, then neutralized with an equal volume of 1 M KHCO_3_ and centrifuged at 3,220 × g for 30 min at 4°C. The resulting supernatants were collected for metabolite determination. Before centrifugation, an aliquot was set aside for TAG determination. Tissue lactate and TAG concentrations were assessed spectrophotometrically using commercial kits (SPINREACT, see above). Glycogen content was quantified following the method of Keppler and Decker [43], which involves enzymatic hydrolysis of glycogen to glucose. Glycogen levels were calculated by subtracting the free glucose concentration from the total glucose measured after enzymatic digestion with amyloglucosidase (Sigma–Aldrich, Ref. A7420). Accordingly, both total and free glucose were quantified using a commercial kit (SPINREACT, see above).

#### 2.5.2. Cortisol Levels

Plasma cortisol concentrations were measured with a commercial enzyme immunoassay kit (Cortisol EIA, DETECTX, K003‐H, ARBOR ASSAYS, NCal International Standard Kit), following the manufacturer’s protocol. Briefly, plasma samples were mixed with two volumes of Dissociation Reagent and diluted with Assay Buffer to fall within the assay’s detection range. Samples and standards were plated in duplicate, followed by the addition of a specific monoclonal antibody and a cortisol‐peroxidase conjugate. Plates were incubated for 1 h at 24°C with vigorous shaking (850 rpm) using a Sky Line shaker‐thermostat (Elmi). After incubation, wells were washed with the provided Wash Buffer, and the chromogenic substrate 3,3^′^, 5,5^′^‐tetramethylbenzidine (TMB) was added. The reaction proceeded for 30 min at 24°C in the dark without agitation. Finally, Stop Solution was added to terminate the reaction, and absorbance was measured at 450 nm.

#### 2.5.3. Activity of Metabolic Enzymes in Plasma

The activities of lactate dehydrogenase (LDH, EC 1.1.1.27; Kit Ref. 1001261), alkaline phosphatase (ALP, EC 3.1.3.1; Kit Ref. 1001131), aspartate aminotransferase (AST, EC 2.6.1.1; Kit Ref. 1001161), and alanine aminotransferase (ALT, EC 2.6.1.2; Kit Ref. 1001171) were determined in plasma using commercial kits from SPINREACT S.A., following the manufacturer’s instructions. Briefly, enzyme activities were calculated from changes in absorbance at baseline and at 1‐min intervals over 3 min, and the mean rate of change (ΔA/min) was determined. For LDH, AST, and ALT, enzyme activity was assessed by monitoring the decrease in nicotinamide adenine dinucleotide (NADH) concentration, whereas for ALP, the formation of *p*‐nitrophenol was measured. In all cases, changes in absorbance were directly proportional to the catalytic activity of the corresponding enzyme. Measurements were performed at an incubation temperature of 37°C and at wavelengths of 340 nm (LDH, AST, and ALT) and 405 nm (ALP). All samples were analyzed in duplicate as described in Section 2.5.1. Enzyme activities were normalized to plasma protein concentration and expressed as mU mg^−1^ protein.

#### 2.5.4. Fatty Acid Analysis

The FA profile of perivisceral fat samples was determined by gas chromatography, following the method described by Rodríguez‐Ruiz et al. [44]. The procedure was performed using a Hewlett‐Packard 4890 Series II gas chromatograph (Hewlett‐Packard Company, Avondale, PA), applying a modified version of the direct transesterification method developed by Lepage and Roy [45], which does not require prior lipid extraction. Nonadecanoic acid (19:0) was used as the FA standard for identification.

#### 2.5.5. Histomorphological and Histochemical Studies in Liver and Intestine

The surrounding adipose and connective tissues of the intestine were carefully removed prior to fixation. Liver and anterior intestine samples were washed in running tap water, dehydrated through a graded ethanol series, cleared in xylene, and embedded in paraffin wax. Sections (6 μm thick) were cut using a rotary microtome and mounted on gelatin‐coated slides. Subsequently, the sections were rehydrated in distilled water and stained with hematoxylin and eosin (H&E) following the protocol of Bancroft and Stevens [46]. The Periodic acid‐Schiff (PAS) and Alcian Blue (AB) staining methods were also applied to detect carbohydrates and glycoproteins, as described by Martoja and Martoja‐Pierson [47].

#### 2.5.6. Morphometric Analyses in Liver and Intestine

For liver samples, hepatocyte area was measured (400×). Hepatocyte selection was based on the following criteria: (i) clear delineation from surrounding cells and (ii) the presence of a visible nucleus, ensuring that a representative portion of the cell or nucleus was sectioned. To minimize selection bias, measurements were conducted in a systematic serpentine pattern, beginning at the upper left‐hand corner of each image and proceeding horizontally back and forth to the lower right corner. All hepatocytes meeting the selection criteria were analyzed sequentially until 30 cells were assessed per fish (120 measurements per experimental group).

For each section of the anterior intestine (examined at 100× magnification), the average width of the mucosal‐submucosal layer thickness (MS, µm) was estimated based on 10 measurements taken between villi at various locations along the section (10 per fish; 40 measurements per experimental group). The surface mucosal length was divided by the submucosal perimeter length (assessed at 20× magnification) to obtain a relativized mucosal‐to‐submucosal surface ratio (MSR), calculated from four opposing measurements per cross‐section (4 per fish; 16 measurements per experimental group). Goblet cell density (GC mm^−2^) was calculated as the mean number of neutral GC counted at 100× magnification across four opposing fields per section (4 per fish; 16 measurements per group). The highest‐quality cross‐section from each specimen was selected for morphological measurements.

Images for morphometric assessment were acquired using a light microscope (Nikon Eclipse Ci‐L) equipped with a Jenoptik ProgRes CT5 camera. Quantitative measurements were conducted using Fiji software (ImageJ2, National Institutes of Health, USA).

#### 2.5.7. Total RNA Isolation and mRNA Expression Analysis

Samples were homogenized with an Ultra‐Turrax T8 homogenizer (IKA‐Werke, Germany), and total RNA was extracted from liver biopsies with the NucleoSpin RNA kit (Macherey‐Nagel, Germany), following the manufacturer’s instructions. Genomic DNA was eliminated by on‐column rDNase digestion, which is included in the protocol. RNA integrity was assessed with a 2100 Bioanalyzer (Agilent Technologies, USA), and RNA concentration was measured with a NanoDrop One spectrophotometer (Thermo Fisher Scientific, USA). Only samples with an RNA Integrity Number (RIN) greater than 8.0 were selected for quantitative real‐time PCR (qPCR). Prior to gene expression assessment, 500 ng of total RNA per sample was reverse transcribed into complementary DNA (cDNA) using the qSCRIPT cDNA Synthesis Kit (Quanta BioSciences, USA) in a final reaction volume of 20 µL. Resulting cDNA samples were diluted 1:10 in nuclease‐free water for subsequent use in qPCR assays.

Quantitative real‐time PCR (qPCR) was performed using a CFX96 Real‐Time PCR Detection System coupled to a C1000 Touch Thermal Cycler (Bio‐Rad Laboratories, USA). Reactions were run in 96‐well white Hard‐Shell PCR plates sealed with Microseal ‘B’ Seals (Bio‐Rad). Each 10 µL reaction contained 5 µL of PerfeCTa SYBR Green FastMix (2×, Quanta BioSciences, USA), 0.5 µL each of forward and reverse primers (final concentration of 200 nM), and 4 µL of 1:10 diluted cDNA (corresponding to 10 ng of input RNA). Gene expression profiling targeted transcripts related to growth regulation and lipid metabolism, including: growth hormone receptor 1 (*ghr1*), growth hormone receptor 2 (*ghr2*), insulin‐like growth factor 1 (*igf1*), fatty acid desaturase 2 (*fads2*), stearoyl‐CoA desaturase 1a (*scd1a*), hepatic lipase (*hl*), lipoprotein lipase (*lpl*), hormone‐sensitive lipase (*hsl*), and adipose triglyceride lipase (*atgl*). Primer sequences and amplification details are provided in Supporting Information: Appendix A. The qPCR thermal cycling protocol was as follows: initial denaturation at 95°C for 10 min, followed by 40 cycles of 95°C for 15 s and 60°C for 30 s. A melting curve analysis was included (from 60–95°C, increasing by 0.5°C per step, with 70 reads) to verify product specificity and rule out primer‐dimer formation. To optimize qPCR conditions for each gene, a pooled cDNA sample was serially diluted (1:10 from 10 ng to 100 fg), and calibration curves were generated. All qPCR reactions showed *R* ^2^ values > 0.980 and efficiencies between 90.0% and 110.0%. Relative gene expression was normalized to beta‐actin (*actb*), selected as a reference gene due to its stability under the experimental conditions (*M*‐value < 0.5). Relative gene expression was calculated using the ∆∆CT method, corrected for amplification efficiency [48], and analyzed using CFX Manager™ software (Bio‐Rad).

### 2.6. Statistical Analysis

All results are expressed as mean ± standard error of the mean (SEM). Prior to statistical evaluation, data were tested for normality and homogeneity of variance using the Kolmogorov‐Smirnov and Levene’s tests, respectively. A two‐way analysis of variance (ANOVA) was applied, considering experimental diet (C+, C‒, and LP) and feeding condition (Cf and Ft) as fixed independent factors. When significant effects were detected (*p* < 0.05), Tukey’s *post hoc* test was applied for multiple comparisons among groups. Statistical analyses and graphical representations were carried out with GraphPad Prism version 8.0 (GraphPad Software, Inc., San Diego, CA, USA).

Principal component analysis (PCA) was used to identify multivariate patterns associated with growth and metabolic responses in *S. aurata*. This approach included plasma and liver variables selected based on physiological relevance and the lowest number of missing values. Pearson correlation coefficients were calculated to assess linear relationships between variables, and significance thresholds were defined as *p*  < 0.05 ( ^∗^), *p*  < 0.01 ( ^∗∗^), *p*  < 0.001 ( ^∗∗∗^), and *p*  < 0.0001 ( ^∗∗∗∗^). A correlation matrix was generated, and a heatmap was used to visualize these associations. Statistical computations and visualizations were carried out using R software (version 4.4.0, released 2024‐04‐24). The libraries “FactoMineR” (https://cran.r-project.org/web/packages/FactoMineR/index.html) and “corrr” (https://cran.r-project.org/web/packages/corrr/index.html) were used for data processing, whereas “Factoextra” (https://cran.r-project.org/web/packages/factoextra/index.html) and “corrplot” (https://cran.r-project.org/web/packages/corrplot/index.html) were used for visualization.

## 3. Results

### 3.1. Growth Performance and Somatic Indices

**Figure 1:**
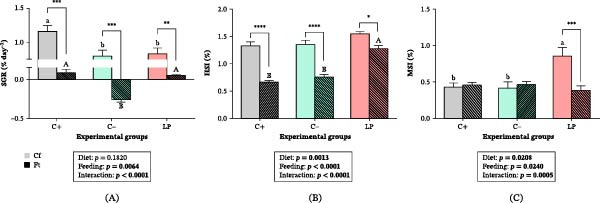
Growth and performance parameters of juvenile gilthead seabream (*Sparus aurata*) fed different experimental diets for 90 days to visual satiety (C+, positive control containing 20% FM and 9% FO; C‒, negative control containing 5% FM and 5% FO; LP, C‒ diet supplemented with 1% *LB-LIVERprotect*, 10 g kg^−1^ feed), followed by two feeding conditions for an additional 14‐day period (Cf, continuous feeding; Ft, fasting). (A) Specific growth rate (SGR, % day^−1^); (B) hepatosomatic index (HSI, %); and (C) mesenteric index (MSI, %). Data are presented as mean ± SEM for eight fish per experimental group. Different letters indicate significant differences among dietary treatments (lowercase for Cf, uppercase for Ft). Asterisks denote significant differences between feeding conditions (Cf vs. Ft), according to two‐way ANOVA followed by Tukey’s *post hoc* test: *p*  < 0.05 ( ^∗^), *p*  < 0.01 ( ^∗∗^), *p*  < 0.001 ( ^∗∗∗^), and *p*  < 0.0001 ( ^∗∗∗∗^). Significant *p*‐Values in bold.

Two‐way ANOVA showed a significant Diet × Feeding condition interaction for SGR, HSI, and MSI (*p*  < 0.001). SGR was significantly higher in the C+ group than in the C‒ and LP groups (*p*  < 0.05; Figure 1A). In fasted fish, SGR decreased in all groups (*p*  < 0.01), with negative values observed only in the C‒ group (*p*  < 0.001). HSI did not differ among Cf treatments (*p*  > 0.05; Figure 1B); however, it decreased significantly in fasted fish from the C+ and C‒ groups (*p*  < 0.0001). The decrease was attenuated in fasted LP fish (*p* < 0.05). Under Cf conditions, MSI was higher in LP fish compared to the C+ and C‒ groups (*p*  < 0.05; Figure 1C). In fasted fish, MSI decreased significantly in the LP group (*p*  < 0.001), reaching values comparable to those observed in fasted C+ and C‒ fish (*p*  > 0.05). No significant differences in MSI between feeding conditions were detected in the C+ and C‒ groups (*p*  > 0.05).

### 3.2. Biochemical Parameters in Plasma and Liver

Table 3 summarizes the metabolic parameters analyzed in plasma and liver. A significant Diet × Feeding condition interaction was detected for plasma glucose (*p*  < 0.05). Under Cf, plasma glucose levels did not differ among diets (*p*  > 0.05). In contrast, fasted fish previously fed plant‐based diets (C‒ and LP) showed higher glucose levels (*p* < 0.05), with a significant increase in the C‒ group compared to its Cf counterpart (*p*  < 0.05). Neither lactate nor plasma protein levels were affected by diet (*p*  > 0.05); however, both parameters decreased during fasting. This reduction was significant for plasma lactate in the C+ and LP groups and for plasma proteins in the C+ and C‒ groups (*p*  < 0.05). Plasma TAG levels were higher in fish fed the LP diet than in the C+ group (*p*  < 0.05), with intermediate values observed in the C‒ group. Under fasting conditions, TAG levels increased in the C‒ group (*p*  < 0.05), decreased in the C+ group (*p*  < 0.05), and remained unchanged in the LP group (*p*  > 0.05), resulting in a significant Diet × Feeding condition interaction (*p*  < 0.05). Plasma cholesterol levels did not differ among diets or feeding conditions (*p*  > 0.05). Plasma cortisol levels were higher in Cf fish from the C‒ group than in the C+ group (*p*  < 0.05), with intermediate values in LP‐fed fish. In fasted fish, cortisol levels increased significantly in all groups (2.70‐fold in C+, 2.32‐fold in C‒, and 2.22‐fold in LP; *p*  < 0.001), with the highest values observed in fasted C‒ fish (*p*  < 0.05).

**Table 3:** Plasma and hepatic biochemistry of juvenile gilthead seabream (*Sparus aurata*) fed different experimental diets for 90 days to visual satiety (C+, positive control containing 20% FM and 9% FO; C‒, negative control containing 5% FM and 5% FO; LP, C‒ diet supplemented with 1% *LB-LIVERprotect*, 10 g kg^−1^ feed), followed by two feeding conditions for an additional 14‐day period (Cf, continuous feeding; Ft, fasting).

Parameters	Experimental groups						*p*‐Value		
	C+		C‒		LP				
	Cf	Ft	Cf	Ft	Cf	Ft	Diet	Feeding	Interaction
**PLASMA**									

Glucose (mM)	4.20 ± 0.20	3.93 ± 0.15^B^	4.01 ± 0.15	5.59 ± 0.40 ^∗^^A^	5.16 ± 0.58	5.29 ± 0.38^A^	**0.0058**	0.0891	**0.0197**
Lactate (mM)	1.66 ± 0.12	1.14 ± 0.15 ^∗^	1.64 ± 0.02	1.22 ± 0.10	1.62 ± 0.08	1.07 ± 0.10 ^∗^	0.7488	**<0.0001**	0.7966
Proteins (mg mL^−1^)	32.11 ± 0.80	28.99 ± 0.69 ^∗^	32.19 ± 0.88	27.29 ± 1.49 ^∗^	32.39 ± 1.92	29.10 ± 1.43	0.7142	**0.0012**	0.7530
Triglycerides (mM)	2.10 ± 0.18^b^	1.52 ± 0.12 ^∗^^C^	3.12 ± 0.29^ab^	4.86 ± 0.46 ^∗^^A^	4.10 ± 0.93^a^	3.30 ± 0.58^B^	**<0.0001**	**0.0137**	**0.0159**
Cholesterol (mg dL^−1^)	309.90 ± 14.95	302.30 ± 18.05	286.70 ± 30.36	318.70 ± 12.02	279.20 ± 35.07	278.20 ± 25.73	0.4463	0.6751	0.6353
Cortisol (ng mL^−1^)	6.81 ± 0.98^b^	18.37 ± 0.82 ^∗∗∗^^B^	13.49 ± 1.99^a^	31.31 ± 1.55 ^∗∗∗∗^^A^	10.22 ± 0.66^ab^	22.66 ± 2.42 ^∗∗∗^^B^	**<0.0001**	**<0.0001**	0.1317
LDH (mU mg^−1^ protein)	7.25 ± 1.22	10.35 ± 2.10	11.67 ± 2.52	12.91 ± 2.13	9.95 ± 1.81	13.31 ± 2.98	0.2756	0.1742	0.8749
ALP (mU mg^−1^ protein)	4.49 ± 0.23	2.33 ± 0.20 ^∗^^AB^	4.16 ± 0.38	2.89 ± 0.24^A^	3.75 ± 0.69	2.18 ± 0.06 ^∗^^B^	**0.0453**	**<0.0001**	0.4441
AST (mU mg^−1^ protein)	0.05 ± 0.02	0.09 ± 0.03	0.05 ± 0.01	0.09 ± 0.02	0.08 ± 0.01	0.08 ± 0.02	0.7983	0.1370	0.5523
ALT (mU mg^−1^ protein)	0.02 ± 0.01	0.01 ± 0.01^B^	0.01 ± 0.01	0.17 ± 0.05 ^∗∗∗^^A^	0.03 ± 0.01	0.05 ± 0.02^B^	**0.0110**	**0.0065**	**0.0044**

**LIVER**									

Glucose (mg g^−1^ ww)	1.26 ± 0.10	2.89 ± 0.19 ^∗∗∗∗^^A^	1.22 ± 0.11	2.34 ± 0.13 ^∗∗∗∗^^B^	1.23 ± 0.08	2.88 ± 0.10 ^∗∗∗∗^^A^	0.0586	**<0.0001**	0.0852
Glycogen (mg g^−1^ ww)	9.81 ± 0.66	4.56 ± 0.25 ^∗∗∗∗^^AB^	10.77 ± 0.63	3.94 ± 0.44 ^∗∗∗∗^^B^	11.51 ± 0.54	5.10 ± 0.17 ^∗∗∗∗^^A^	**0.0361**	**<0.0001**	0.2042
Triglycerides (mg g^−1^ ww)	85.85 ± 3.57^b^	40.22 ± 2.56 ^∗∗∗∗^^B^	106.10 ± 2.18^a^	34.98 ± 3.99 ^∗∗∗∗^^B^	104.78 ± 1.58^a^	60.99 ± 0.85 ^∗∗∗∗^^A^	**<0.0001**	**<0.0001**	**<0.0001**

*Note:* Data are presented as mean ± SEM for eight fish per experimental group. Different superscript letters indicate significant differences among dietary treatments (lowercase for Cf, uppercase for Ft). Asterisks denote significant differences between feeding conditions (Cf vs. Ft), according to two‐way ANOVA followed by Tukey’s *post hoc* test: *p* < 0.05( ^∗^), *p* < 0.01 ( ^∗∗^), *p* < 0.001 ( ^∗∗∗^), and *p* < 0.0001 ( ^∗∗∗∗^). Significant *p*‐Values in bold.

Abbreviations: ALP, alkaline phosphatase; ALT, alanine aminotransferase; AST, aspartate aminotransferase; LDH, lactate dehydrogenase; ww, wet weight.

The activities of LDH and AST did not vary among diets or feeding conditions (*p*  > 0.05). ALP activity was similar among Cf fish (*p*  > 0.05); however, fasting reduced ALP activity across all groups, with significant decreases observed only in the C+ and LP groups (*p*  < 0.05). Fasted fish from the C‒ group showed higher ALP activity compared to fasted fish from the C+ and LP groups (*p*  < 0.05). A significant Diet × Feeding condition interaction was detected for plasma ALT (*p*  < 0.01). ALT activity did not differ among diets under Cf (*p*  > 0.05), whereas a marked increase was observed in fasted C‒ fish (17‐fold increase; *p*  < 0.001).

No differences in hepatic glucose or glycogen concentrations were detected among Cf fish (*p*  > 0.05). By contrast, hepatic glucose concentrations increased in fasted fish in all groups (2.29‐fold in C+, 1.92‐fold in C‒, and 2.34‐fold in LP; *p*  < 0.0001), with higher values observed in the C+ and LP groups and lower values in the C‒ group (*p*  < 0.05). Hepatic glycogen content decreased in all fasted fish (54% in C+, 63% in C‒, and 56% in LP; *p*  < 0.0001), with the lowest concentrations detected in fasted C‒ fish and the highest in fasted LP fish (*p*  < 0.05). A significant Diet × Feeding condition interaction was detected for hepatic TAG concentrations (*p*  < 0.0001). Under Cf, hepatic TAG concentrations were higher in fish fed the C‒ and LP diets (*p*  < 0.05). However, in fasted fish, hepatic TAG concentrations decreased in all groups (53% in C+, 67% in C‒, and 42% in LP; *p*  < 0.0001), with higher concentrations remaining in the LP group (*p*  < 0.05).

### 3.3. Fatty Acid Analysis of Perivisceral Fat

The FA profile of perivisceral fat varied with diet and feeding condition (Table 4). Polyunsaturated fatty acids (PUFA) were the predominant FA class, followed by monounsaturated fatty acids (MUFA) and saturated fatty acids (SFA). SFA levels were significantly higher in the C+ group under both feeding conditions than in the C‒ and LP groups (ΣSFA, myristic acid [14:0], and palmitic acid [16:0]; *p*  < 0.05). Total MUFA did not differ among Cf fish (*p*  > 0.05). In fasted fish, MUFA levels increased in the C‒ (*p*  < 0.01) and LP groups (*p*  < 0.05), with higher values in C‒ compared to C+ and LP (*p*  < 0.05). PUFA content was higher in fish fed plant‐based diets under both feeding conditions (C‒ and LP; *p*  < 0.05). A significant Diet × Feeding condition interaction was detected for linoleic acid (LA, 18:2n‐6) and α‐linolenic acid (ALA, 18:3n‐3) (*p*  < 0.05). LA was the most abundant PUFA and showed higher levels in the C‒ and LP groups than in the C+ group, regardless of feeding condition (*p*  < 0.05). In fasted fish, LA levels decreased in the C‒ group (*p*  < 0.05) but remained higher, together with values in the LP group, than those in the C+ group (*p*  < 0.05). ALA levels were higher in fish fed PP‐ and VO‐rich diets (C‒ and LP) than in the C+ group (*p*  < 0.05). In fasted fish, ALA remained higher in the C‒ group but decreased in the LP group (*p*  < 0.01), with the lowest levels observed in the C+ group (*p*  < 0.05). A significant Diet × Feeding condition interaction was detected for EPA (*p*  < 0.01). EPA (20:5n‐3) levels were higher in the LP group and lower in the C‒ group (*p*  < 0.05); in fasted LP fish, EPA levels decreased (*p*  < 0.01), whereas the highest values were observed in the C+ group (*p*  < 0.05). Arachidonic acid (ARA, 20:4n‐6) and DHA (22:6n‐3) showed a similar pattern, with higher levels in the C+ group under both feeding conditions and lower levels in the C‒ and LP groups (*p*  < 0.05). However, under Cf conditions, this trend was observed for ARA but did not reach statistical significance (*p*  > 0.05).

**Table 4:** Fatty acid composition (% of total fatty acids) in perivisceral fat of juvenile gilthead seabream (*Sparus aurata*) fed different experimental diets for 90 days to visual satiety (C+, C‒, and LP), followed by two feeding conditions for an additional 14‐day period (Cf, continuous feeding; Ft, fasting).

Parameters	Experimental groups						*p*‐Value		
	C+		C‒		LP				
	Cf	Ft	Cf	Ft	Cf	Ft	Diet	Feeding	Interaction
14:0	2.76 ± 0.12^a^	2.63 ± 0.07^A^	1.68 ± 0.12^b^	1.80 ± 0.07^B^	1.57 ± 0.05^b^	1.97 ± 0.04 ^∗∗^^B^	**<0.0001**	0.7348	**0.0204**
16:0	16.19 ± 0.52^a^	15.45 ± 0.46^A^	12.72 ± 0.29^b^	12.51 ± 0.19^B^	13.37 ± 0.99^ab^	13.49 ± 0.27^B^	**0.0110**	0.6982	0.6533
18:0	3.94 ± 0.04	3.97 ± 0.08	3.65 ± 0.14	3.89 ± 0.14	3.86 ± 0.12	3.73 ± 0.06	0.1791	0.5824	0.1984
16:1n‐7	5.12 ± 0.18^a^	5.01 ± 0.12^A^	3.71 ± 0.19^b^	4.09 ± 0.06^B^	3.35 ± 0.06^b^	3.73 ± 0.06 ^∗^^C^	**<0.0001**	0.6901	0.0859
16:2n‐4	0.59 ± 0.03^a^	0.57 ± 0.02^A^	0.39 ± 0.02^b^	0.44 ± 0.01^B^	0.44 ± 0.01^b^	0.35 ± 0.01^C^	**<0.0001**	0.5551	**0.0054**
16:3n‐4	0.53 ± 0.02^a^	0.47 ± 0.01 ^∗^^A^	0.31 ± 0.01^b^	0.29 ± 0.00^C^	0.34 ± 0.01^b^	0.35 ± 0.01^B^	**<0.0001**	0.6638	**0.0** **199**
18:1n‐9	23.70 ± 0.77	24.62 ± 0.43^C^	25.44 ± 0.48	27.56 ± 0.24 ^∗∗^^A^	23.87 ± 0.12	26.28 ± 0.40 ^∗^^B^	**0.0002**	**0.0091**	0.2345
18:2n‐6 (LA)	21.14 ± 0.33^c^	20.12 ± 0.28^B^	33.43 ± 0.11^a^	30.65 ± 0.55 ^∗^^A^	30.04 ± 0.06^b^	29.40 ± 0.45^A^	**<0.0001**	0.8538	**0.0481**
18:3n‐3 (ALA)	2.27 ± 0.07^b^	2.27 ± 0.06^C^	3.62 ± 0.21^a^	3.27 ± 0.10^A^	3.60 ± 0.04^a^	2.92 ± 0.10 ^∗∗^^B^	**<0.0001**	0.2780	**0.0283**
18:4n‐3	0.59 ± 0.04	0.66 ± 0.03^A^	0.60 ± 0.02	0.59 ± 0.04^AB^	0.66 ± 0.04	0.51 ± 0.00 ^∗^^B^	0.0599	0.4528	**0.0175**
20:1n‐9	1.21 ± 0.10	1.24 ± 0.04^A^	0.88 ± 0.08	1.21 ± 0.06 ^∗^^A^	1.10 ± 0.06	0.99 ± 0.02^B^	**0.0027**	0.0938	**0.0325**
20:4n‐6 (ARA)	0.57 ± 0.00	0.57 ± 0.00^A^	0.50 ± 0.04	0.45 ± 0.01^B^	0.55 ± 0.02	0.47 ± 0.00^B^	0.0848	0.2893	0.1362
20:4n‐3	0.31 ± 0.02	0.39 ± 0.01 ^∗∗^	0.34 ± 0.01	0.37 ± 0.02	0.39 ± 0.06	0.38 ± 0.03	0.2775	**0.0312**	0.1244
20:5n‐3 (EPA)	3.04 ± 0.07^ab^	3.16 ± 0.14^A^	2.70 ± 0.05^b^	2.54 ± 0.14^B^	3.30 ± 0.08^a^	2.24 ± 0.10 ^∗∗^^B^	**0.0008**	0.1538	**0.0** **028**
22:5n‐3	1.38 ± 0.02	1.57 ± 0.01 ^∗^^A^	1.02 ± 0.10	1.05 ± 0.06^B^	1.02 ± 0.02	0.98 ± 0.08^B^	**0.0002**	0.7839	0.3919
22:6n‐3 (DHA)	9.52 ± 0.29^a^	9.17 ± 0.62^A^	5.03 ± 0.28^b^	4.31 ± 0.13 ^∗^^B^	5.43 ± 0.27^b^	4.80 ± 0.22^B^	**<0.0001**	0.8516	0.8770
Other FA	6.30 ± 0.53	6.01 ± 0.19^A^	3.65 ± 1.67	2.82 ± 0.14^B^	4.91 ± 0.00	5.66 ± 0.14 ^∗^^A^	0.0710	0.8717	0.5140
ΣSFA	22.89 ± 0.67^a^	22.05 ± 0.60^A^	18.05 ± 0.46^b^	18.21 ± 0.35^B^	18.29 ± 0.32^b^	19.19 ± 0.30^B^	**<0.0001**	0.9105	0.2069
ΣMUFA	31.14 ± 0.19	33.03 ± 0.63^B^	31.95 ± 0.46	34.92 ± 0.31 ^∗∗^^A^	30.03 ± 0.08	32.78 ± 0.48 ^∗^^B^	**0.0002**	**0.0005**	0.4849
ΣPUFA	39.18 ± 0.74^b^	37.85 ± 0.35^B^	46.96 ± 0.08^a^	43.32 ± 0.70 ^∗^^A^	46.64 ± 0.42^a^	41.67 ± 0.87 ^∗^^A^	**0.0001**	**0.0114**	0.1114
Σn‐3	17.22 ± 0.40^a^	16.58 ± 0.58^A^	13.04 ± 0.07^c^	12.16 ± 0.49^B^	15.87 ± 0.70^b^	11.80 ± 0.59 ^∗^^B^	**<0.0001**	0.1445	**0.0313**
Σn‐6	21.67 ± 0.36^c^	20.69 ± 0.31^B^	33.88 ± 0.15^a^	31.17 ± 0.57 ^∗^^A^	31.45 ± 0.25^b^	29.87 ± 0.43^A^	**<0.0001**	0.7811	0.1485
Σn‐9	24.91 ± 0.87	25.86 ± 0.47^B^	26.32 ± 0.51	28.76 ± 0.16 ^∗∗^^A^	24.85 ± 0.10	27.25 ± 0.23 ^∗∗^^A^	**0.0132**	0.4259	**0.0157**
n‐3/n‐6	0.78 ± 0.00^a^	0.83 ± 0.03^A^	0.40 ± 0.02^c^	0.39 ± 0.01^B^	0.49 ± 0.00^b^	0.40 ± 0.02 ^∗∗^^B^	**<0.0001**	0.9183	**0.00** **85**
EPA/DHA	0.33 ± 0.01^b^	0.35 ± 0.01^B^	0.57 ± 0.03^a^	0.62 ± 0.02^A^	0.66 ± 0.06^a^	0.43 ± 0.03 ^∗^^B^	**<0.0001**	0.6348	**0.0007**

*Note:* Data are presented as mean ± SEM for eight fish per experimental group. Additional details are described in the legend of Table 3.

Abbreviations: ALA, α‐linolenic acid; ARA, arachidonic acid; DHA, docosahexaenoic acid; EPA, eicosapentaenoic acid; FA, fatty acids; LA, linoleic acid; MUFA, monounsaturated fatty acids; PUFA, polyunsaturated fatty acids; SFA, saturated fatty acids.

Two‐way ANOVA revealed a significant Diet × Feeding condition interaction for total n‐3 FA, total n‐9 FA, the n‐3/n‐6 ratio, and the EPA/DHA ratio (*p*  < 0.05). Total n‐3 FA levels were highest in the C+ group, lowest in C‒, and intermediate in LP‐fed fish under both feeding conditions (*p*  < 0.05); however, in fasted LP fish, n‐3 levels decreased significantly (*p*  < 0.05). Total n‐6 FA levels were highest in fish fed the C‒ diet, followed by LP and C+ groups (*p*  < 0.05). In fasted fish, n‐6 levels decreased in the C‒ group (*p*  < 0.05) but remained higher, together with LP, than those in the C+ group (*p*  < 0.05). The n‐3/n‐6 ratio was highest in the C+ group, intermediate in LP, and lowest in C‒ (*p*  < 0.05). In fasted fish, the ratio decreased in LP‐fed fish (*p*  < 0.01), resulting in similarly lower values in the LP and C‒ groups compared to C+ (*p*  < 0.05). In contrast, the EPA/DHA ratio was higher in the C‒ and LP groups than in the C+ group (*p*  < 0.05); however, in fasted LP fish, this ratio decreased (*p*  < 0.05), reaching values similar to those observed in the C+ group. Finally, n‐9 FA levels did not differ among Cf fish (*p*  > 0.05) but increased significantly in fasted fish previously fed plant‐based diets (C‒ and LP; *p*  < 0.01), exceeding those observed in the C+ group.

### 3.4. Histomorphological and Histochemical Analysis of the Liver and Intestine

**Figure 2:**
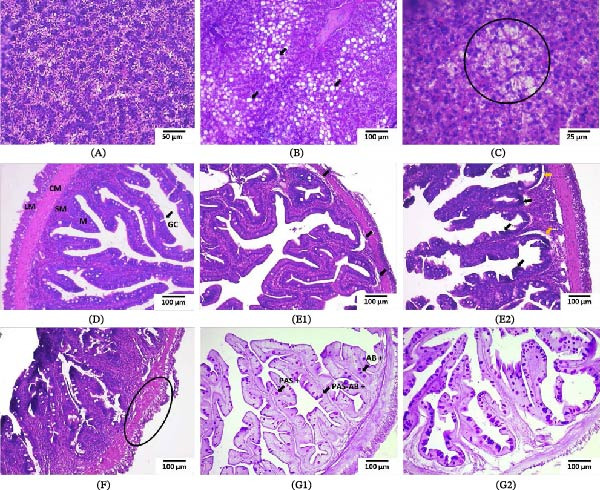
Representative photomicrographs of the liver (L) and anterior intestine (AI) of juvenile gilthead seabream (*Sparus aurata*) fed different experimental diets for 90 days to visual satiety (C+, C‒, and LP), followed by two feeding conditions for an additional 14‐day period (Cf, continuous feeding; Ft, fasting). (A) Normal histological architecture of L from fish fed the C+ diet (H&E); (B) L from fish fed the C‒ diet, showing a high number of unilocular adipocytes (black arrows) (H&E); (C) L from fasted fish in the LP group, showing multilocular adipocytes (circle) (H&E); (D) normal histological architecture of AI from fish fed the C+ diet (LM, longitudinal muscle; CM, circular muscle; SM, submucosa layer; M, mucosa layer; GC, goblet cells) (H&E); (E1) AI from fish fed the C‒ diet, showing subepithelial spaces between mucosa and submucosa layers (black arrows) (H&E); (E2) AI from fasted fish in the C‒ group, showing marked subepithelial spaces (yellow arrows), loss of normal villi architecture, and mucosal hyperchromatism (black arrows) (H&E); (F) AI from fasted fish in the C‒ group, showing alterations in the tunica muscularis (circle) (H&E); (G1) AI from fish fed the C+ diet, showing goblet cells (black arrows) containing neutral mucins (PAS^+^), acidic mucins (AB^+^), and mixed mucins (PAS‐AB^+^); and (G2) AI from fasted fish in the LP group, showing an increased number of goblet cells (PAS‐AB).

**Figure 3:**
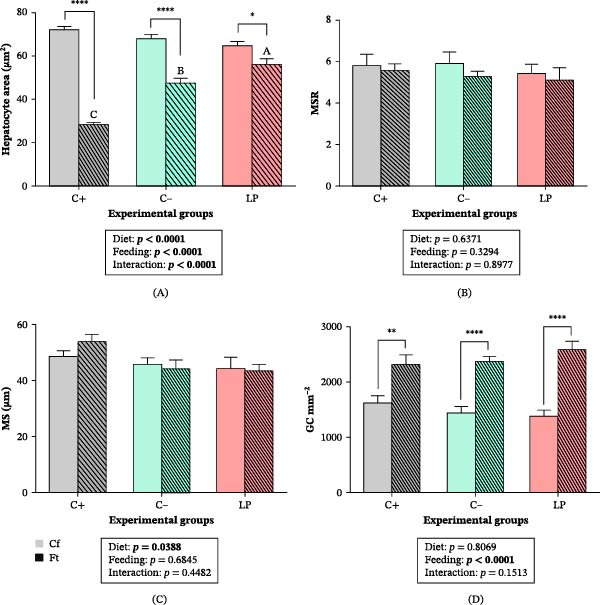
Intestinal and hepatic morphometric analyses of juvenile gilthead seabream (*Sparus aurata*) fed different experimental diets for 90 days to visual satiety (C+, C‒, and LP), followed by two feeding conditions for an additional 14‐day period (Cf, continuous feeding; Ft, fasting). (A) Hepatocyte area (µm^2^); (B) mucosal‐to‐submucosal surface ratio (MSR); (C) mucosal‐submucosal layer thickness (MS, µm); and (D) goblet cells (GC mm^−2^). Data are presented as mean ± SEM for four fish (different measurements) per experimental group. Additional details are described in the legend of Figure 1.

In the C+ group, the liver displayed normal histological architecture (Figure 2A). In contrast, fish from the C‒ and LP groups showed a high abundance of adipocytes (unilocular fat cells) surrounding the intrahepatic exocrine pancreas, with a lower abundance observed in the LP group (Figure 2B,C). A significant Diet × Feeding condition interaction was detected for hepatocyte area (*p*  < 0.0001). Hepatocyte atrophy was observed in fasted fish in all groups (*p*  < 0.05), with larger hepatocyte areas observed in fasted LP fish (*p*  < 0.05; Figure 2C; Figure 3A).

Fish fed the C+ diet showed normal intestinal histological architecture (Figure 2D). In the C‒ group, fish under both feeding conditions developed subepithelial spaces, characterized by separation between the mucosal and submucosal layers (Figure 2E1,E2); this alteration was not observed in the LP group. In addition, alterations in the tunica muscularis were observed in fasted fish in all dietary groups, including separation of the inner and outer muscle layers and the presence of cavities within the circular muscle layer (Figure 2F). MSR and MS (Figure 3B,C) were not affected by diet or feeding condition (*p*  > 0.05). GC density increased in fasted fish across all groups (*p*  < 0.01), with the most pronounced increases observed in the C‒ and LP groups (*p*  < 0.0001; Figure 2G1,G2; Figure 3D).

### 3.5. Gene Expression in Liver

**Figure 4:**
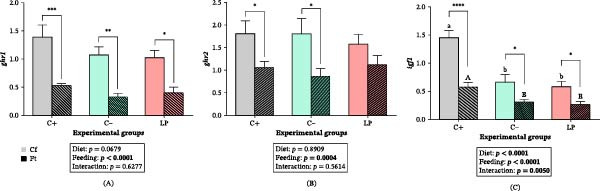
Relative hepatic expression (∆∆CT) of genes involved in growth and endocrine regulation in juvenile gilthead seabream (*Sparus aurata*) fed different experimental diets for 90 days to visual satiety (C+, C‒, and LP), followed by two feeding conditions for an additional 14‐day period (Cf, continuous feeding; Ft, fasting). (A) *ghr1*; (B) *ghr2*; and (C) *igf1*. Data are presented as mean ± SEM for eight fish per experimental group. Additional details are described in the legend of Figure 1.

Neither *ghr1* nor *ghr2* expression differed among diets (*p*  > 0.05; Figure 4A,B). The expression of both genes was reduced in fasted fish in all groups (*p*  < 0.05), although the decrease in *ghr2* was not significant in fasted LP fish (*p*  > 0.05). A significant Diet × Feeding condition interaction was detected for *igf1* expression (*p*  < 0.01; Figure 4C). Under both feeding conditions (Cf and Ft), *igf1* expression was lower in the C‒ and LP groups than in the C+ group (*p*  < 0.05). Under fasting conditions, *igf1* expression decreased in all groups (*p*  < 0.05), with the strongest reduction observed in the C+ group (*p*  < 0.0001).

**Figure 5:**
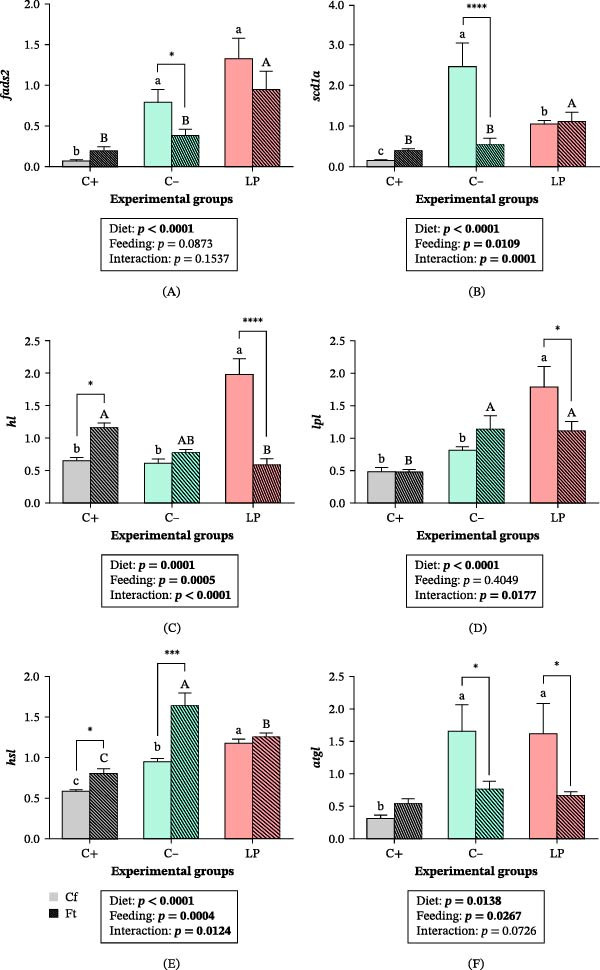
Relative hepatic expression (∆∆CT) of genes involved in lipid metabolism in juvenile gilthead seabream (*Sparus aurata*) fed different experimental diets for 90 days to visual satiety (C+, C‒, and LP), followed by two feeding conditions for an additional 14‐day period (Cf, continuous feeding; Ft, fasting). (A) *fads2*; (B) *scd1a*; (C) *hl*; (D) *lpl*; (E) *hsl*; and (F) *atgl*. Data are presented as mean ± SEM for eight fish per experimental group. Additional details are described in the legend of Figure 1.

Among genes involved in lipid metabolism, *fads2* expression was higher in the C‒ and LP groups (*p*  < 0.05; Figure 5A). In fasted fish, *fads2* expression remained highest in the LP group, whereas it decreased significantly in the C‒ group (*p*  < 0.05), reaching levels comparable to those of the C+ group. A significant Diet × Feeding condition interaction was detected for *scd1a* expression (*p*  < 0.001). The expression of *scd1a* was higher in fed C‒ fish (*p*  < 0.05) and decreased in fasted C‒ fish (*p*  < 0.0001; Figure 5B). In contrast, fasted LP fish maintained *scd1a* expression at levels similar to those of fed LP fish (*p*  > 0.05) and showed higher expression than the fasted C+ and C‒ groups (*p*  < 0.05). A significant Diet × Feeding condition interaction was also detected for *hl* and *lpl* expression (*p*  < 0.05). The expression patterns of both genes were similar, showing higher transcript levels in the LP group (*p*  < 0.05). In fasted LP fish, *hl* expression decreased markedly (*p*  < 0.0001), reaching the lowest levels among all fasted groups, whereas the fasted C+ group showed comparatively higher expression (*p*  < 0.05; Figure 5C). Although *lpl* expression also decreased significantly in fasted LP fish (*p*  < 0.05), it remained higher, together with the C‒ group, compared to fasted C+ fish (*p*  < 0.05; Figure 5D). A significant Diet × Feeding condition interaction was detected for *hsl* expression (*p*  < 0.05), with higher expression in LP fish under Cf, followed by the C‒ and C+ groups (*p*  < 0.05; Figure 5E). In fasted fish, *hsl* expression increased in the C‒ (*p*  < 0.001) and C+ groups (*p*  < 0.05), with the highest levels observed in the C‒ group and the lowest in the C+ group (*p*  < 0.05). In the LP group, *hsl* expression did not differ between feeding conditions (Cf and Ft; *p*  > 0.05). Expression of *atgl* was higher in the C‒ and LP groups (*p*  < 0.05) but decreased in fasted fish after 14 days (*p*  < 0.05; Figure 5F). No differences in *atgl* expression were detected between Cf and Ft fish in the C+ group or among fasted groups (*p*  > 0.05).

### 3.6. Multivariate Analysis

**Figure 6:**
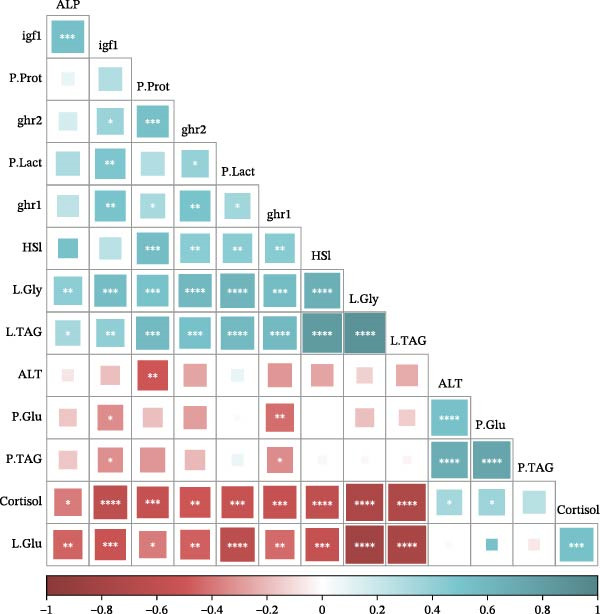
Correlation matrix of growth, metabolic, and stress‐related parameters measured in plasma and liver of juvenile gilthead seabream (*Sparus aurata*) fed different experimental diets for 90 days to visual satiety (C+, C‒, and LP), followed by two feeding conditions for an additional 14‐day period (Cf, continuous feeding; Ft, fasting). Abbreviations: ALP, alkaline phosphatase; ALT, alanine aminotransferase; *ghr1*, growth hormone receptor 1; *ghr2*, growth hormone receptor 2; Glu, glucose; Gly, glycogen; HSI, hepatosomatic index; *igf1*, insulin‐like growth factor 1; L, liver; Lact, lactate; P, plasma; Prot, proteins; TAG, triglycerides. Asterisks indicate the level of significance: *p*  < 0.05 ( ^∗^), *p*  < 0.01 ( ^∗∗^), *p*  < 0.001 ( ^∗∗∗^), and *p*  < 0.0001( ^∗∗∗∗^).

The correlation matrix is shown in Figure 6. Liver glycogen and TAG concentrations showed significant positive correlations with each other and with HSI, plasma protein and lactate, plasma ALP activity, and hepatic expression of *ghr1*, *ghr2*, and *igf1*. Most of these variables were also significantly and positively intercorrelated. In contrast, hepatic glucose and plasma cortisol concentrations were significantly and positively correlated with each other, and both variables showed negative correlations with most of the remaining parameters. Plasma glucose, TAG, and cortisol concentrations, as well as ALT activity, were positively correlated with one another, although the correlation between TAG and cortisol was not significant. Additional negative correlations were observed between the expression of both *ghr1* and *igf1* and plasma glucose and TAG concentrations, as well as between ALT activity and plasma protein concentration.

**Figure 7:**
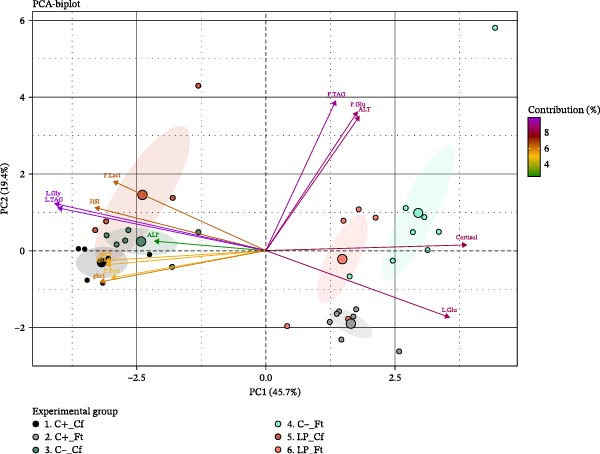
Principal component analysis (PCA) illustrating multivariate response patterns related to growth, metabolism, and stress response based on key variables measured in plasma and liver of juvenile gilthead seabream (*Sparus aurata*) fed different experimental diets for 90 days to visual satiety (C+, C‒, and LP), followed by two feeding conditions for an additional 14‐day period (Cf, continuous feeding; Ft, fasting). Abbreviations are as described in Figure 6. Small dots represent individual values, whereas large dots represent the mean of each experimental group. Ninety‐five percent confidence ellipses are shown for each experimental group.

To explore multivariate patterns among the measured variables, a PCA was applied using somatic indices and biochemical and molecular parameters from plasma and liver that varied across experimental conditions (Figure 7). The variables included HSI; plasma glucose, lactate, TAG, protein, cortisol, ALP, and ALT; and hepatic glucose, glycogen, TAG, and the expression of *ghr1*, *ghr2*, and *igf1*. The PCA explained 65.1% of the total variance, with the first principal component (PC1) accounting for 45.7% and the second principal component (PC2) for 19.4%. PC1 was mainly driven by plasma cortisol and hepatic glucose, together with hepatic glycogen and TAG concentrations, HSI, and, to a lesser extent, the hepatic expression of *ghr1*, *ghr2*, and *igf1*. PC2 was primarily defined by plasma TAG, glucose, and ALT activity. The PCA score plot showed a clear separation between Cf and Ft fish along the horizontal axis. Cf fish clustered on the left side of the plot, whereas Ft fish were distributed toward the right. Under fasting conditions, LP fish clustered between the C+ and C‒ groups, while the C‒ group showed a broader dispersion along both principal components.

## 4. Discussion

The present study shows that dietary supplementation with a plant extract‐ and microalgae‐based nutraceutical modulates metabolic and tissue‐level responses in *S. aurata* juveniles exposed to a combination of plant‐based diets and feed deprivation. By comparing a conventional formulation with a diet rich in PP and VO, and the same formulation supplemented with *LB-LIVERprotect*, the results indicate that nutraceutical supplementation may contribute to improved physiological resilience under combined nutritional stress conditions. These effects were reflected in somatic indices, biochemical parameters, and molecular biomarkers, supporting the use of functional dietary strategies to mitigate nutritional and metabolic disturbances in aquaculture species.

### 4.1. Modulation of Hepatic Lipid Metabolism Under Nutritional Challenges

At the hepatic level, diets rich in plant‐based ingredients were associated with lipid redistribution, which was differentially modulated by nutraceutical supplementation under both feeding and fasting conditions. The number of adipocytes surrounding the intrahepatic exocrine pancreas increased in fish fed the C‒ and LP diets, regardless of feeding status, in agreement with previous reports describing similar effects in fish fed VO‐rich diets [39, 49]. However, this increase was less pronounced in the LP group, indicating a coordinated metabolic adjustment that may contribute to liver protection. In parallel, LP‐fed fish showed higher MSI values, suggesting that excess lipids derived from plant‐based diets were preferentially stored as perivisceral fat rather than accumulating in hepatic tissue. In fasted fish, MSI values significantly decreased in the LP group, consistent with the mobilization of mesenteric lipid reserves while preserving hepatic glycogen and lipid levels within a healthy range, comparable to those observed in the C+ group. Together, these responses suggest that nutraceutical supplementation promotes a more balanced lipid partitioning strategy, potentially protecting fish from fasting‐associated metabolic disturbances.

The modulation of lipid partitioning observed at the hepatic level was further supported by changes in the regulation of key genes involved in lipid metabolism. Hepatic expression of *lpl* and *hl*, encoding two enzymes central to lipid absorption, storage, and mobilization [50], varied in parallel with changes in perivisceral fat deposition. These enzymes facilitate FA export toward peripheral tissues such as adipose depots [51, 52]. In line with this, their enhanced expression in LP‐fed fish was associated with increased MSI values, supporting preferential lipid export and storage in mesenteric tissue under continuous feeding. The increased *lpl* expression observed in LP‐fed fish is consistent with previous studies in teleosts fed diets rich in PP, VO, or lipids [51, 53, 54], as well as with reports describing protective effects against fatty liver development [55]. Conversely, the significant reduction in MSI in fasted LP fish was accompanied by lower transcript levels of both genes, indicating a reduced hepatic contribution to lipid trafficking and a shift toward the utilization of perivisceral reserves. Additionally, under fasting conditions, an inverse regulation of *lpl* and *hl* was detected across all groups, a response previously reported in *S. aurata* during feed deprivation [50]. This pattern likely reflects a coordinated mechanism to secure energy supply during periods of nutrient shortage [53].

Differences in lipid mobilization strategies among dietary groups were further evidenced by changes in the gene expression patterns of lipolytic enzymes involved in hepatic lipid catabolism. Atgl and Hsl, which act sequentially in the breakdown of stored lipids [50, 52], showed increased expression in fish continuously fed the C‒ and LP diets. This was possibly due to a preventive response to limit excessive hepatic lipid accumulation associated with plant‐based formulations [51, 56–58]. Under fasting conditions, hepatic *atgl* expression markedly decreased in both groups, suggesting a reduced reliance on hepatic lipid stores and a preferential mobilization of mesenteric fat reserves, particularly in fasted LP fish [59]. In contrast, *hsl* expression increased in fasted fish, as observed in other teleosts [51], with the strongest response observed in the C‒ group, followed by LP group. The compensatory pattern may reflect the coregulation of lipolytic pathways in response to reduced *atgl* expression [56] and was associated with increased circulating TAG levels and marked depletion of hepatic TAG reserves (67%) in C‒ fish. Collectively, these findings indicate a higher metabolic cost of fasting in fish fed plant‐based diets, an effect that appears partially alleviated by nutraceutical supplementation.

Fasting was also associated with marked structural alterations in the liver, although these effects were partially attenuated by nutraceutical supplementation. Fasted fish from all experimental groups exhibited significant hepatocyte atrophy, consistent with previous observations in various teleost species subjected to feed deprivation [60, 61]. This cellular shrinkage is in agreement with the reduction in HSI commonly reported in fasted fish, including gilthead seabream [62–67]. However, both hepatocyte atrophy and the decline in HSI were less pronounced in the LP‐fasted group, indicating a better preservation of hepatic energy status. Given that hepatocyte volume reduction is closely associated with glycogen depletion [36, 68, 69], the attenuated atrophy observed in fasted LP fish suggests that higher residual energy reserves were maintained compared to C+ and C‒. This pattern is consistent with the idea that *LB-LIVERprotect* supplementation contributes to buffering hepatic energy loss under nutritional stress.

Changes in liver condition were further associated with alterations in endocrine signaling related to growth and energy balance. HSI showed a positive correlation with the hepatic expression of both growth hormone receptors (*ghr1* and *ghr2*), reinforcing the link between liver metabolic status and Gh‐mediated regulation. Gh exerts its effects through binding to specific receptors (Ghr) in target tissues, thereby stimulating insulin‐like growth factor 1 (Igf1) production and promoting somatic growth via the Gh/Igf axis [70]. While hepatic *ghr1* and *ghr2* expression was not markedly affected by diet, *igf1* expression was downregulated in fish continuously fed plant‐based diets (C‒ and LP). These results suggest that nutraceutical supplementation did not enhance *igf1* expression and therefore did not improve growth performance under feeding conditions [39]. Similar reductions in hepatic *igf1* expression have been reported in gilthead seabream fed low FM diets or under limited protein availability, which reduces hepatic sensitivity to Gh [71, 72]. Fasting was associated with a clear endocrine shift toward a catabolic state. This was evidenced by the reduced expression of *ghr1* and *ghr2* across all experimental groups, consistent with previous observations in gilthead seabream [71, 73] and other teleosts [66, 74–76] subjected to feed deprivation. Reduced receptor expression is likely to diminish hepatic responsiveness to Gh and may be associated with lower *igf1* expression, contributing to growth suppression during periods of nutrient shortage [62, 76, 77]. In line with this, *igf1* expression decreased in fasted fish, as has also been previously reported in teleosts [62, 63, 66, 75, 77–79], including gilthead seabream [80]. The decrease was positively correlated with *ghr* expression and negatively correlated with circulating and hepatic metabolic indicators. This reflects a fasting‐induced metabolic imbalance that reduces hepatic sensitivity to Gh and favors the mobilization of lipid reserves [81]. However, these fasting‐associated endocrine alterations appeared to be partially attenuated in fish fed the LP diet, as the reduction in *ghr* expression was less pronounced and not significant for *ghr2*. The moderated response may have contributed to maintaining positive specific growth rates (SGR > 0) in LP‐fasted fish, in contrast to the negative SGR observed in the C‒ group. Together, these results indicate that the fasting protocol was sufficient to induce growth impairment in all experimental groups. Interestingly, supplementation of plant‐based diets with *LB-LIVERprotect* helped partially mitigate some endocrine and growth‐related consequences of feed deprivation without overriding diet‐driven growth constraints.

### 4.2. Stress Response and Liver Integrity

Additionally, fasting was associated with a higher metabolic cost in fish fed plant‐based diets, particularly those receiving the unsupplemented formulation, as reflected by indicators of an intensified stress response and increased energy mobilization. The lower SGR values and greater metabolic effort to cope with feed deprivation observed in C‒ may be linked to the inhibitory effects of elevated cortisol on somatic growth through increased energy expenditure [82, 83]. Under stressful conditions, metabolic energy is reallocated from growth toward the maintenance of homeostasis [84]. In this context, the C‒ group showed higher plasma cortisol levels under both feeding and fasting conditions, as previously reported [39], indicating a sustained primary stress response. Multivariate analysis further supported this pattern, revealing increased depletion of hepatic energy reserves in fasted fish, as evidenced by negative correlations with hepatic glycogen and TAG levels [64, 85]. This was accompanied by elevated plasma glucose, lactate, and TAG concentrations in C‒ fasted fish, characteristic of the secondary stress response [86]. Reduced plasma protein levels in the C‒ group may additionally reflect increased protein catabolism to support hepatic gluconeogenesis [37]. In contrast, LP‐fasted fish displayed a metabolic response closer to that of the control group, as shown by PCA clustering. This clustering was largely influenced by plasma cortisol levels, as well as by the maintenance or depletion of hepatic energy reserves. Accordingly, these results suggest that *LB-LIVERprotect* supplementation mitigated several diet‐ and fasting‐associated stress responses, as reflected by lower cortisol levels, reduced reliance on hepatic energy reserves, and a more stable plasma metabolic profile during feed deprivation.

Changes in plasma enzyme activities further indicated that fasting‐induced metabolic stress differentially affected liver integrity depending on diet formulation. Plasma enzymes such as AST, ALT, and ALP are widely used as markers of tissue damage, particularly hepatic dysfunction, due to their release into the bloodstream following cellular injury [87–89]. In the present study, PP‐based diets did not alter basal enzyme activities under feeding conditions, in agreement with previous reports [90]. However, fasting was associated with marked changes that were clearly diet‐dependent. A significant increase in ALT activity was observed exclusively in fasted fish from the C‒ group, whereas values remained stable in C+ and LP‐fasted fish. Similar ALT elevations have been reported in teleosts exposed to prolonged stressors, including feed restriction and environmental challenges [87, 89, 91–93]. Notably, only fasted C‒ fish exhibited an ALT:AST ratio greater than 1 (ALT > AST), a condition commonly associated with severe hepatic damage [89]. This ratio was below 1 in fish receiving the nutraceutical‐supplemented diet. In parallel, ALP activity decreased in all fasted fish, consistent with previous observations under fasting conditions [65]. However, C‒ fish retained higher ALP values under fasting. Although changes in ALP activity may reflect alterations in membrane transport or biliary function [91, 94], the overall enzyme profile is consistent with a higher degree of liver impairment in fasted C‒ fish. By contrast, nutraceutical supplementation appeared to attenuate these alterations, in line with previous studies reporting hepatoprotective effects of functional dietary compounds in teleosts [95].

### 4.3. Perivisceral Fatty Acid Composition and Hepatic Desaturase Regulation

Diet formulation and nutraceutical supplementation substantially influenced FA composition in perivisceral fat. In farmed fish, dietary changes are known to alter FA profiles in several tissues [96], particularly in mesenteric fat, which acts as an energy storage tissue. Although increased MUFA content has been reported in fish fed plant‐based diets [97], this effect was not observed among the experimental groups in the present study. However, reductions in SFA content and increases in LA, ALA, and total PUFA in fish fed diets rich in PP and VO, as well as in those supplemented with microalgae, are consistent with previous findings in gilthead seabream [6, 98]. Notably, nutraceutical supplementation was linked to a more favorable FA profile in LP‐fed fish, characterized by increased n‐3 and reduced n‐6 content, along with an improved n‐3/n‐6 ratio in perivisceral fat. Similar effects have been reported in algae‐fed *Chelon labrosus* [99, 100]. In addition, EPA levels were significantly higher in LP‐fed fish, exceeding those of the control group, and increasing the EPA/DHA ratio, likely linked, at least in part, to the contribution of microalgae‐derived FA present in the nutraceutical compound [101]. Regarding fasting effects on FA composition, they were not as pronounced as those observed with diet composition.

The dietary composition and fasting‐induced changes in FA profiles were closely associated with the hepatic regulation of key desaturase enzymes. The liver plays a central role in lipid metabolism, and the expression of desaturases is particularly relevant in fish fed plant‐based diets [29, 50]. The desaturase *fads2*, which is essential for n‐3 LC‐PUFA biosynthesis [102], was upregulated in fish fed C‒ and LP diets, likely in response to dietary lipid composition, particularly the inclusion of VO [103]. This upregulation was associated with higher PUFA levels in the perivisceral fat of these groups. In contrast, *scd1a*, a key enzyme involved in MUFA synthesis and lipid storage regulation [50, 104], showed higher expression in fish fed the C‒ diet, which also exhibited elevated hepatic TAG levels and increased adipocyte abundance. Excessive *scd1a* upregulation has been associated, in other contexts, with lipotoxic effects and hepatic metabolic dysfunction [104], a condition that appeared to be partially attenuated by nutraceutical supplementation. Under fasting conditions, desaturase expression (both *fads2* and *scd1a*) was significantly downregulated only in the C‒ group, as has been observed in previous studies under feed deprivation [52, 79, 105]. The limited desaturase activity may contribute to a reduced capacity of C‒ fish to bioconvert C18 fatty acid precursors into long‐chain PUFA such as EPA and DHA, as previously reported [50, 52]. Although nutraceutical supplementation was associated with higher basal EPA levels in LP‐fed fish, EPA concentrations decreased significantly during fasting. This reduction may be related to the absence of dietary lipid supply during the 14‐day fasting period, together with a moderate (1.4‐fold) but nonsignificant reduction in *fads2* expression, which could have constrained the hepatic conversion capacity toward EPA. Overall, these findings suggest that while the LP diet promotes higher basal EPA availability, prolonged feed deprivation under PP‐ and VO‐rich dietary conditions can still significantly impact fish FA profiles.

### 4.4. Intestinal Histomorphological Responses to Dietary and Fasting Stress

Intestinal histomorphology further reflected the combined effects of diet composition, fasting, and nutraceutical supplementation on tissue integrity and adaptive capacity. Diets rich in PP and VO, particularly when combined with feed deprivation, are known to impair intestinal barrier function and nutrient absorption [106]. In the present study, the formation of subepithelial spaces was evident in fish fed the C‒ diet under both feeding and fasting conditions, consistent with previous reports using the same formulations [39]. Interestingly, in that study, unlike this one, these alterations were also observed in LP‐fed fish, though to a lesser extent. The difference between the two studies may be related to the additional 14‐day feeding period with the nutraceutical‐enriched diet (LP), which may contribute to enhanced medium‐term intestinal barrier integrity. Comparable improvements have been described in gilthead seabream fed microalgae‐enriched diets that support tight junction stability [107]. By contrast, fish fed the unsupplemented C‒ diet did not show a comparable improvement, suggesting that prolonged exposure to this formulation may contribute to the persistence of alterations in mucous cell dynamics and intestinal structure, particularly when combined with fasting. However, further studies are required to disentangle the relative contributions of feeding duration, diet composition, fasting, and nutraceutical supplementation to these intestinal responses.

Fasting was associated with structural changes in the tunica muscularis across all groups, including separation of muscle layers and cavity formation in the circular muscle. These alterations may be related to fluctuations in intestinal length and diameter during feeding‐fasting cycles [61] or to immune responses triggered by fasting [108]. In parallel, an increase in goblet cell (GC) numbers was observed in fasted fish, independent of diet type, suggesting that this response was primarily driven by fasting rather than by differences in diet composition. This finding is in agreement with previous studies reporting increased GC abundance during feed deprivation in several fish species [109, 110]. However, other studies have described a reduction in GC density following longer fasting periods than those applied in the present study [111, 112]. In the intestine, mucus plays a key role in maintaining epithelial hydration and barrier function. Enhanced mucus production and the continuous shedding of mucus and cellular debris into the lumen may facilitate the recycling of endogenous material that can be reused as an energy source during fasting [113]. Therefore, the observed rise in GC numbers may reflect an adaptive intestinal response aimed at maintaining epithelial integrity and supporting metabolic homeostasis during periods of nutrient deprivation.

Despite the relevance of the present findings, which can be considered exploratory, some limitations should be acknowledged. FA composition and gene expression regulation were not jointly assessed in both hepatic tissue and mesenteric fat, which would have provided additional insight into tissue‐specific lipid metabolism. The fasting protocol applied represents a specific nutritional challenge, and exploring different fasting durations or more severe feed deprivation scenarios would help to define the robustness and scope of the observed responses. Furthermore, the absence of a refeeding phase after fasting limits the evaluation of recovery capacity and the reversibility of fasting‐induced hepatic alterations. The study was conducted using a single inclusion level of the nutraceutical, although future dose‐response studies would help to better define the optimal level of supplementation. Finally, the absence of a control diet supplemented with the nutraceutical prevents the isolation of its sole effects under optimal dietary conditions. Addressing these aspects in future studies would further clarify the mechanisms of action and the practical relevance of this nutraceutical in aquaculture.

## 5. Conclusions

In a previous study, the effects of *LB-LIVERprotect* on growth, metabolism, welfare, and intestinal integrity were evaluated after 90 days of feeding, showing that the nutraceutical did not reverse most of the adverse nutritional effects associated with plant‐based raw materials [39]. In the present study, however, following a subsequent fasting challenge, the results suggest that *LB-LIVERprotect* may confer relevant physiological advantages during periods of feed deprivation. Specifically, *LB-LIVERprotect* supplementation was associated with improvements in lipid metabolism, reduced signs of fatty liver development, enhanced mobilization of mesenteric fat relative to hepatic reserves, and a more favorable FA profile. In addition, *LB-LIVERprotect* appeared to attenuate indicators of liver damage associated with both plant‐based diets and fasting, as reflected by reduced hepatocyte atrophy and lower plasma activities of enzymes indicative of hepatic damage. These effects were accompanied by reduced plasma cortisol levels in LP fish during fasting, consistent with the preservation of hepatic energy reserves and potentially contributing to aquaculture‐relevant performance metrics, such as positive SGR values. However, the intestine does not appear to be a primary target tissue of *LB-LIVERprotect* in terms of restoring functionality after dietary changes or feed deprivation. Given the increasing use of plant‐based ingredients and the strategic application of fasting periods in modern aquaculture, this nutraceutical may represent a relevant tool to support fish health, metabolic stability, and production sustainability.

## Author Contributions

**Anyell Caderno**: investigation, methodology, formal analysis, data curation, visualization, writing – original draft, writing – review and editing. **Milagrosa Oliva**: investigation, formal analysis, visualization, writing – review and editing. **Alba Galafat**: methodology, formal analysis, data curation. **Antonio Astola**: methodology, formal analysis, data curation. **Francisco Javier Alarcón-López**: resources, funding acquisition, writing – review and editing. **Juan Miguel Mancera**: resources, funding acquisition, writing – review and editing. **Juan Antonio Martos-Sitcha**: conceptualization, investigation, resources, funding acquisition, project administration, writing – review and editing, supervision.

## Funding

This work was cofinanced by the European Union through the 2014–2020 ERDF Operational Programme and by the Department of Economic Transformation, Industry, Knowledge, and Universities of the Regional Government of Andalusia (project reference: FEDER‐UCA18‐107182). Additional funding was provided by the University of Almería spin‐off LifeBioencapsulation S.L., and the study was partially supported by the Spanish Ministry of Science and Innovation (project IRSAF, Grant PID2020‐117557RB‐C22). Anyell Caderno was supported by a Ph.D. fellowship from the Regional Government of Andalusia (2021; reference: PREDOC_02015).

## Conflicts of Interest

The authors declare no conflicts of interest.

## Supporting Information

Additional supporting information can be found online in the Supporting Information section.

## Supporting information


**Supporting Information** Appendix A. Primer sequences used for qPCR.

## Data Availability

Data are available upon request from the authors.
