# Supplementary material for: Fasting Reveals the Effects of a Plant Extract‐ and Microalgae‐Derived Nutraceutical on Lipid Metabolism and Hepatic Physiology in Juvenile Sparus aurata Fed a Plant‐Based Diet
Source: Aquac Nutr. 2026 May 19;2026:9345086. doi: 10.1155/anu/9345086 (PMC13189500; doi:10.1155/anu/9345086)
Supplement: Supplementary file 1 — Supporting Information Appendix A. Primer sequences used for qPCR. [file ANU-2026-9345086-s001.docx]

**Appendix A.** Primer sequences used for qPCR.

| Gene | Sequence 5’‒3’ | R^2^ and E | Accession number | Amplicon size (bp) |
| --- | --- | --- | --- | --- |
| *actb* | **F:** TCTTCCAGCCATCCTTCCTCG | R^2^ = 0.999  E = 97.2% | X89920.1 | 108 |
|  | **R:** TGTTGGCATACAGGTCCTTACGG |  |  |  |
| *ghr1* | **F:** ACCTGTCAGCCACCACATGA | R^2^ = 0.999  E = 101.9% | AF438176.2 | 99 |
|  | **R:** TCGTGCAGATCTGGGTCGTA |  |  |  |
| *ghr2* | **F:** GAGTGAACCCGGCCTGACAG | R^2^ = 0.998  E = 90.0% | AY573601.2 | 75 |
|  | **R:** GCGGTGGTATCTGATTCATGGT |  |  |  |
| *igf1* | **F:** GCCACACCCTCTCACTACTG | R^2^ = 0.982  E = 108.5% | EF563837.1 | 196 |
|  | **R:** AAGCAGCACTCGTCCACA |  |  |  |
| *fads2* | **F:** GCAGGCGGAGAGCGACGGTCTGTTCC | R^2^ = 0.991  E = 94.5% | MN061683.1 | 72 |
|  | **R:** AGCAGGATGTGACCCAGGTGGAGGCAGAAG |  |  |  |
| *scd1a* | **F:** CGGAGGCGGAGGCGTTGGAGAAGAAG | R^2^ = 0.990  E = 105.4% | JQ277703.1 | 199 |
|  | **R:** AGGGAGACGGCGTACAGGGCACCTATATG |  |  |  |
| *hl* | **F:** TTGTAGAAGGTGAGGAAAAC | R^2^ = 0.993  E = 107.4% | EU254479.1 | 131 |
|  | **R:** GCTCTCCATCAGACCATCC |  |  |  |
| *lpl* | **F:** CGTTGCCAAGTTTGTGACCTG | R^2^ = 0.992  E = 96.3% | AY495672.2 | 192 |
|  | **R:** AGGGTGTTCTGGTTGTCTGC |  |  |  |
| *hsl* | **F:** GCTTTGCTTCAGTTTACCACCATTTC | R^2^ = 0.997  E = 102.5% | EU254478.1 | 122 |
|  | **R:** GATGTAGCGACCCTTCTGGATGATGTG |  |  |  |
| *atgl* | **F:** GTGCTTCAGTCCTGGATGTCTTC | R^2^ = 0.991  E = 94.8% | JX975711.1 | 93 |
|  | **R:** AGCCTTGCAGGTCCATGTTGA |  |  |  |
